# Association between WHO First-Step Analgesic Use and Risk of Breast Cancer in Women of Working Age

**DOI:** 10.3390/ph16020323

**Published:** 2023-02-20

**Authors:** Hyun Sook Oh, Hwa Jeong Seo

**Affiliations:** 1 Department of Applied Statistics, School of Social Science, Gachon University, Seongnam-si 13120, Gyeinggi-do, Republic of Korea; 2 Medical Informatics and Health Technology (MiT), Department of Health Care Management, College of Social Science, Gachon University, Seongnam-si 13120, Gyeinggi-do, Republic of Korea

**Keywords:** breast neoplasms, occupational health, age groups, economic status, analgesics

## Abstract

We assessed the association between breast cancer and analgesic use in women of a specific working-age group. The Korean National Health Insurance Service–National Sample Cohort database (KNHIS–NSC) data were analyzed. We calculated hazard ratios (HRs) with 95% confidence intervals (CIs) for patients’ cancer risk based on whether the women participated in economic activity (PEA or not PEA (NPEA) groups) and analgesic use. Additionally, breast cancer incidence variations by age group, and PEA or NPEAs, health behavior, Charlson Comorbidity Index, and analgesic use were evaluated. The PEA group had a higher cancer risk than the NPEA group (HR = 1.542, 95% CI: 1.345–1.768, *p* < 0.001). Breast cancer risk was high in the PEA, high income, and no history of exercise groups, but significantly reduced in the regular-use-of-analgesics group. Notably, the working age group of 40~49 years, within the PEA group, had the highest HR of breast cancer development (HR = 1.700, 95% CI = 1.361–2.124, *p* < 0.001); whereas regular analgesic use in those aged 25~39 years decreased breast cancer risk (HR = 0.611, 95% CI = 0.427–0.875, *p* < 0.05). In conclusion, our results suggest that individuals at a high-risk of comorbidity may benefit from regular use of analgesics, which may prove to be a useful strategy for breast cancer prevention in the Young-aged group.

## 1. Introduction

The participation rate of women in economic activities in the Organization for Economic Co-operation and Development (OECD) member countries increased from 57.0% in 1991 to 63.6% in 2016 [1]. Meanwhile, although the incidence of major cancers has continuously decreased, the incidence of breast cancer has increased by 4.5% between 2005 and 2016 in South Korea [2]. In the United States, approximately two-thirds of breast cancer patients are aged ≥55 years [3]; however, in Korea, it is most common among women aged 40~49 years (approximately 33.4%) and <40 years (13%) [4]. In Asia, including Korea, the incidence rate of breast cancer among women aged 40–49 years is more than twice as high as that in Western countries [4]. Given the high incidence of breast cancer among women aged 40~49 years in Korea, studies have been conducted on the influence of income level and social participation on such increasing occurrence of breast cancer in this population [5]. Yoo et al. projected that the incidence of breast cancer in Korea could continuously increase due to the westernized environment and increased participation of women in society [6].

The proportion of women participating in economic activities in Korea has constantly increased from 47.4% in 2003 to 50.8% in 2017. There are variations in the pattern of participation in economic activities among Korean women depending on the age group. Between the ages of 25~39, pattern of participation in economic activities (including employment rate) shows a decrease due to marriage, childbirth, and childcare; increase between ages 40~49, when women return to work; and significant decrease again in women over the age of 50 [7]. Since the characteristics of social participation differ by age group, it is necessary to examine the risk of cancer development according to economic activity in each age group.

Furthermore, economic-activity-related stress and chronic inflammation have a causal relationship with breast cancer incidence among women [8]. In a study on occupational stress and mental health of working women who are the heads of a household, 46.7% had poor mental health and were diagnosed with hypertension (7.3%) and obesity (2.1%) [9]. Although employed women have high levels of sociopsychological stress [10,11], they are less likely than their non-employed counterparts to exhibit health prevention behavior [12] and cancer prevention practices [13]. Women’s participation in economic activities, alongside household work and childrearing, has negative influences on their health [14]. Obesity, hypertension, diabetes, and stress lead to chronic inflammation, which increases the risk of cancer. Meanwhile, analgesics relieve inflammation and reduce this risk [15]. Consequently, analgesic drugs, such as acetaminophen and non-steroidal anti-inflammatory drugs (NSAIDs) have been investigated as cancer chemo-preventive agents in experimental and observational studies [15,16,17].

However, few studies have assessed the cancer-inhibiting effect of analgesics, especially among working age groups with different characteristics of social participation, nor whether economic activity and analgesic use are related to breast cancer development. 

Thus, this study aimed to analyze the association between economic activity, regular analgesic use, and breast cancer development in women of specific age groups, to provide a basis for improving cancer prevention practices.

## 2. Results

### 2.1. Participant Characteristics

The participant characteristics are presented in Table 1. In the Young-aged group, 950 (39.7%) were family members of a self-employed person, 815 (34.1%) were dependents of an employee-insured person, and 405 (16.9%) were employee-insured; 875 (36.6%) had a middle income, 820 (34.3%) had a high income, and 695 (29.1%) had a low income. Regarding the health behavior variables, 1820 women (76.2%) had a low body mass index (BMI; <25 kg/m^2^), and 1531 women (64.1%) had no alcohol intake. For the Charlson Comorbidity Index (CCI) score, 1515 (63.4%), 573 (24.0%), and 94 (3.9%) had scores of 0, 1, and ≥3, respectively. Regarding analgesics use, 1572 (65.8%) were non-regular users, 536 (22.4%) were non-users, and 282 (11.8%) were regular users of analgesics.

In the Middle-aged group, 1125 (43.9%) were family members of the self-employed, 825 (32.2%) were dependents of the employee-insured, and 400 (15.6%) were self-employed; In this group 1000 (39.1%) had a high income, 817 (31.9%) had a low income, and 743 (29.1%) had a middle income. Regarding the health behavior variables, 1756 women (68.6%) had a low BMI (<25 kg/m^2^), and 2220 women (86.7%) had low total cholesterol (<240 mg/dL). For the CCI score, 1392 (54.4%), 641 (25.0%), and 226 (8.8%) had scores of 0, 1, and ≥3, respectively. Regarding analgesics use, 1630 (63.7%) were non-regular users, 600 (23.4%) were non-users, and 330 (12.9%) were regular users of analgesics.

In the Senior-aged, 940 (52.7%) were dependents of employee-insured, 500 (28.0%) were family members of self-employed, and 295 (15.6%) were self-employed; 679 (38.0%) had a high income, 611 (34.2%) had a low income, and 495 (27.7%) had a middle income. Regarding the health behavior variables, 1026 women (57.5%) had a low BMI (<25 kg/m^2^), and 945 women (52.9%) never exercised (Never). For the CCI score, 639 (35.8%), 268 (26.2%), and 385 (21.6%) had scores of 0, 1, and ≥3, respectively. Regarding analgesics use, 1013 (56.8%) were non-regular users, 473 (26.5%) were non-users, and 299 (16.8%) were regular users of analgesics (Table 1).

### 2.2. Risk Factors Associated with Cancer Development

#### 2.2.1. Hazard Ratios (HRs) Associated with Cancer Development

The economically active group (PEA group) had a higher risk of breast cancer than the inactive group (NPEA group) (HR = 1.542, 95% confidence interval [CI]: 1.345–1.768, *p* < 0.001). The risk of breast cancer was higher in the high-income group than in the low-income group (HR = 3.089, 95% CI: 2.647–3.605, *p* < 0.001). The risk of breast cancer was lower in participants who reported exercise than in those who did not report exercise (HR = 0.836, 95% CI: 0.751–0.931, *p* < 0.05). It was also lower in participants with regular analgesic use compared to those with no analgesic use (HR = 0.748, 95% CI: 0.614–0.912, *p* < 0.05) (Table 2).

#### 2.2.2. Cumulative Incidence According to Analgesic Use

**Figure 1:**
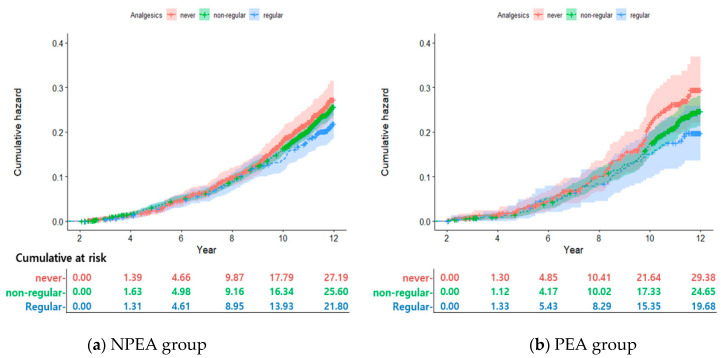
Cumulative incidence of breast cancer according to analgesics use.

In the NPEA group, the 2- and 12-year cumulative incidence rates of breast cancer for never-users of analgesics were 1.4% and 27.2%, respectively. For non-regular users of analgesics, the rates were 1.6% and 25.6%, respectively. For regular analgesic users, the rates were 1.3% and 21.8%, respectively (Figure 1a).

**Figure 2:**
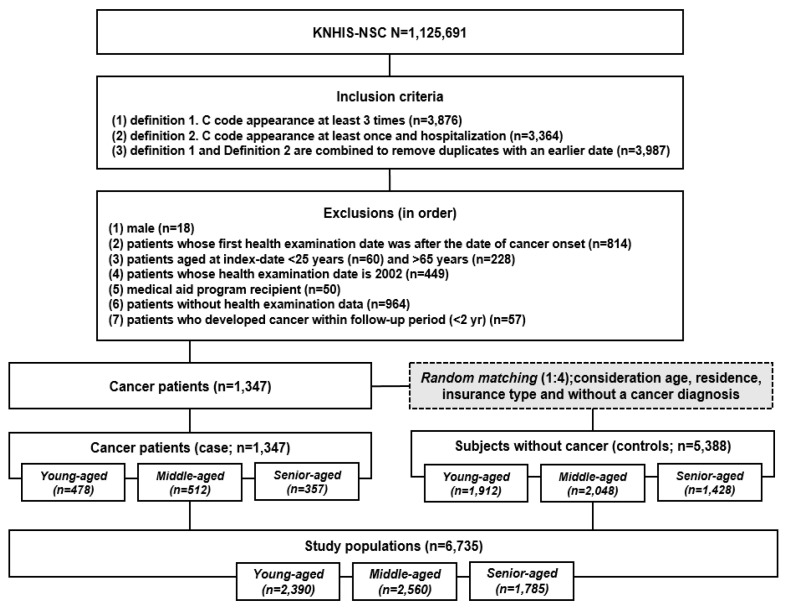
Flow diagram of study population selection.

In the PEA group, the 2- and 12-year cumulative incidence rates of breast cancer for never-users of analgesics were 1.3% and 29.4%, respectively; for non-regular users, 1.1% and 24.7%, respectively; and for regular analgesic users, 1.3% and 19.7%, respectively (Figure 2).

### 2.3. Association between Cancer and Analgesic Use by Working-Age

Among the patients in the Young-aged group (25~39 years), the risk of breast cancer was higher in the PEA group than in the NPEA group (HR = 1.482 95% CI: 1.185–1.854, *p* < 0.05). The HR of those at the middle income level was 1.731 (95% CI: 1.314–2.281, *p* < 0.001, while that of high level was 3.161 (95% CI: 2.415–4.138, *p* < 0.001). The HR of CCI score = 1 was 1.234 (95% CI: 0.994–1.534, *p* < 0.1), CCI score = 2 was 1.555 (95% CI: 1.149–2.105, *p* < 0.05), and CCI scores ≥3 was 2.095 (95% CI: 1.422–3.086, *p* < 0.001). Compared with no analgesic use, regular use was associated with a lower risk of breast cancer (HR = 0.611, 95% CI: 0.427–0.875, *p* < 0.05).

Among the patients in the Middle-aged group (40~49 years), compared with the non-working group, the working group had a significantly higher risk of breast cancer (HR = 1.700, 95% CI: 1.361–2.124, *p* < 0.001). Among the patients in the Senior-aged group (50~64 years), those who participated in economic activity had a significantly higher risk of breast cancer (HR = 1.386, 95% CI: 1.049–1.833, *p* < 0.05), compared with those with no economic activity. Regular analgesic use (HR = 0.741, 95% CI: 0.517–1.061, *p* < 0.1) was associated with a significantly lower risk of breast cancer (Table 3).

## 3. Discussion

Our main findings are as follows. First, the participation in economic activity (PEA) group had a higher risk of breast cancer than the non-PEA (NPEA) group. In particular, there was a notable difference in the cancer risk of women in specific working-age groups, based on their characteristics of social participation. Those in the Middle-aged group (40~49 years) who participated in economic activity had the highest risk of developing breast cancer.

Long working hours adversely affect the health of female workers because working-age women are also involved in other responsibilities such as housework and childcare [14]. The analysis of the female employment rate by age in the Korean Labor & Income Panel Study [18] showed that female employment was concentrated in the age groups of 25~34 years and 45~54 years. In South Korea, the female employment rate is highest (at 69.7%) among those aged 45~49 years [18]. Women in this age group who engage in economic activities have the highest incidence of breast cancer [2], and are more likely than men to be non-regular or daily workers [7]. A 2012 survey of the working hours of women in South Korea showed that those in the Middle-aged group had the highest rate of working 60 h or more per week at 19.8% [7].

Stress is known to be higher in the PEA group than in the NPEA group. Stress increases the level of chronic inflammation, and chronic inflammation can lead to cancer development [19]. Stress and chronic inflammation, factors detrimental to health, have both direct and indirect effects on the incidence of cancer [8]. Yoo et al. [20] reported higher levels of stress in breast cancer patients than in the control patients, and the risk of developing breast cancer was 3.19 times higher in the bad stress-relief group than in the good stress-relief group.

Not only preventative health behaviors such as exercise, but also cancer prevention practices were found to be lower in the PEA group than in the NPEA group [12,13]. Through these results, policy measures are required to increase the practice of disease prevention among women who are participating in society with multiple roles such as work, housework, and childcare, and to detect diseases through early screening.

Although the survival rate of breast cancer has significantly increased with the development of medical technology, the incidence rate of breast cancer is high in premenopausal women in South Korea. Thus, policies are also needed to alleviate the burden of unstable work and childcare for women in the 40~49 age group.

The second finding was the significant association between the use of analgesics and the risk of breast cancer. Regular analgesic use was associated with a lower risk of developing cancer. The use of NSAIDs such as aspirin and ibuprofen has been reported to reduce the risk of cancer and mortality [21]. In a multivariate regression model, regular use of ibuprofen was shown to result in a 48% decreased risk of lung cancer mortality [15]. In addition, long-term use of acetaminophen has been reported to lower the incidence of prostate cancer [22]. The effect of NSAIDs use observed in a meta-analysis was associated with a reduced risk of breast cancer in most studies, regardless of design or case type (events or deaths) [23]. NSAIDs have been reported to reduce breast cancer risk by 20% [24]. Another meta-analysis reported no statistically significant association between ibuprofen and other NSAIDs [25]. This negative study reported that it may have been confounded by reproductive factors [23,25,26]. In the current study, among participants in the economically active group, the 3- and 12-year cumulative incidence rates of breast cancer were higher in the no-analgesic users than in the regular analgesic users (3.6% and 29.6%, respectively, versus 2.2% and 21.1%, respectively). Long-term regular use of analgesics decreased the cumulative incidence of breast cancer. In this study we have indicated an inverse association of use of NSAIDs with risk of breast cancer.

Among the Young-aged group (25~39 years), working and high-risk comorbidity (CCI) were associated with a higher risk of cancer, and regular use of analgesics was associated with a lower risk. Epidemiological studies have repeatedly indicated an association of high-risk comorbidity (CCI), particularly obesity, hypertension and diabetes, with risk of breast cancer [6,27,28]. In Korea, the proportion of breast cancer patients aged 25~39 years and 40~49 years is higher than in the Western countries [4,29]. Breast cancer in young women under 35 years has a high biological malignancy and a poor prognosis [30]. In the Young-aged group, high-risk comorbidity is associated with cancer, suggesting that the management of risk groups diagnosed with obesity, diabetes, and hypertension is necessary.

In addition, we confirmed that regular use of analgesics in the Young-aged group (25~39 years) has an inhibitory effect on cancer development. The drug effect of analgesics was more significant in relatively young women than in older women. Aspirin is an anti-inflammatory analgesic that is known to be effective in preventing myocardial infarction and stroke, and lowering the mortality rate due to cancer [31,32]. Recently, *The Journal of the American Medical Association* (JAMA) published guidelines that stated that aspirin should not be taken, even at low doses, because it leads to an increased risk of gastrointestinal and cerebral hemorrhage in those aged over 60 years [33]. However, it is recommended that participants under the age of 60, who have a ≥10% risk of developing heart disease, take aspirin following consultation with their doctor [34]. As such, there is a possibility that the use of analgesics may have age-related side effects (or antagonism). However, this result should be interpreted with caution.

This study has the following limitations. First, we did not consider occupation type, business category, and work duration. Second, selection bias may exist since economic activity (PEA or NPEA) is classified by insurance type. Third, confounding variables that increase the risk of breast cancer, such as family history, childbirth status, and contraceptive use, were not controlled. Fourth, in the case of alcohol intake and smoking, intensity, quantity, and duration are important, not the experience, such as ever or never [35]. However, in this study, the amount of alcohol and smoking was not considered. In addition, the alcohol intake and smoking in this study have limitations as it is self-reported data. These data need to be interpreted with care, as there may be distortions in answers about drinking, especially smoking, due to the cultural characteristics of Asian women (answers that are not honest). Fifth, since C-reactive protein (CRP) and specific interleukin levels are not registered in the nationwide-based claims data (analyzed data source), we could not reflect these variables as direct parameters of cancer risk. Sixth, although the long-term use of analgesics was analyzed, over-the-counter (OTC) use was not considered in the analysis.

However, despite these limitations, this study is significant in that it analyzed the association between the use of analgesics and the occurrence of breast cancer in working women by classifying them into Young-, Middle-, and Senior-aged groups. Since there may be differences in the cancer prevention effect of non-opioid analgesics (acetaminophen and NSAIDs) depending on the mechanism of action of the analgesic, care should be taken in interpretation. In the future, it is expected that additional studies will be needed in consideration of the limitations of this study.

## 4. Materials and Methods

### 4.1. Study Design and Data Collection

Population-based cohort data was used from the Korean National Health Insurance Service–National Sample Cohort database (KNHIS–NSC) for the period 2002–2013 [36]. Based on this cohort database, we extracted study populations required for analysis using the nested case-control study (or the case-control in a cohort study), and independently followed up cohorts for each age group. This is a useful study design to explore the effects of drug use after drug exposure in patients with newly diagnosed cancer [37,38].

In this study, sampling was carried out as follows: Cancer was defined as the presence of the same C code (ICD10 code; D05, D48.6, D79.80, C50) more than three times [27] (Definition 1; n = 3876) and one or more occurrences of the C code for patients who were hospitalized (Definition 2; n = 3364). Definitions 1 and 2 are combined to remove duplicates with an earlier date (n = 3987).

Patients with cancer before the index date (n = 814) and males were excluded (n = 18) from this study. Furthermore, patients aged <25 years (n = 60) and >65 years (n = 228) at the index date were excluded. Since the CCI is calculated based on the medical information one year before the date of the first health examination, patients whose date of first health examination was in 2002 were excluded (n = 449). Medical aid program recipients whose economic activity (PEA or NPEA) status could not be confirmed (n = 50), or who had missing health examination data were also excluded (n = 964). To select newly diagnosed cancer patients during the cohort monitoring period, patients with a follow-up period <2 years were excluded (n = 57) [39].

We followed up factors influencing cancer incidence based on independent cohorts of three groups: Young-aged, Middle-aged and Senior-aged cohorts. Participants who were not diagnosed with breast cancer for each age cohort were selected in a 1:4 ratio, considering their residence (metropolitan or non-metropolitan area) and insurance type. There were 2390, 2560, and 1785 participants in the Young-aged, Middle-aged, and Senior-aged cohorts, respectively. Overall, 6735 participants were included in the analysis (Figure 2).

### 4.2. Criteria and Definitions

**Figure 3:**
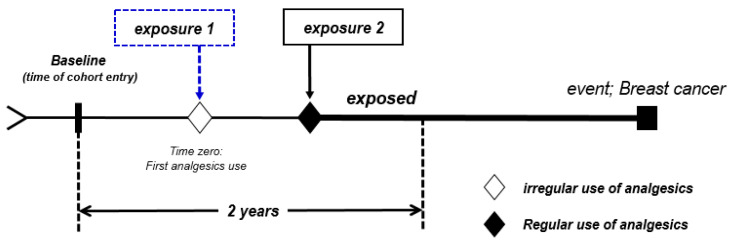
Study design including follow-up period from cohort entry to event occurrence.

In this study, age (25~39, 40~49 and 50~64 years), economic activity status (insurance type, income quantile), health behavior (BMI, alcohol intake, exercise), CCI, and analgesics prescription behavior were analyzed. The baseline age, economic activity variables corresponding to the time of entry into the cohort, and health behavior variables, were based on the date of the first health examination. CCI was calculated based on the medical records, one year prior to the date of the first health examination. Analgesic use (regularity) was calculated from prescription records from the time of cohort entry to the 2-year follow-up (Figure 3).

Age was categorized into three groups (Young-aged, 25–39; Middle-aged, 40–49; and Senior-aged, 50–64 years) based on economic participation; social participation characteristics were different among the groups. In the Senior-aged group (<65 years; ages eligible for employment insurance), we examined the characteristics of the elderly who were economically active. The economic activity variables included insurance type (self-employed, family members of self-employed, employee-insured, dependents of employee) and income quantile (low, 1st–3rd quantile; middle, 4th–6th quantile; and high, 7th–10th quantile) [40]. Economic activity was used to examine the differences between participating in economic activity (PEA) and not PEA (NPEA) groups. To analyze the impact of economic activity, participants were divided into NPEA (non-participating in economic activity group; family members of self-employed and dependents of employee-insured) and PEA groups (participating in economic activity group; self-employed and employee-insured). Income quintiles were classified based on the income of the insured household. The health behavior variables were evaluated according to BMI (kg/m^2^; <25 or ≥25), total cholesterol (mg/dL; <240 or ≥240) [41], self-reported alcohol intake (never: never drinking; ever: drinking 1~7 days a week, rarely, or often), self-reported smoking (never: never smoking; ever: smoked in the past, or currently smoking), and exercise (never: never exercise; ever: exercise 1~7 days a week, rarely, or often) [42]. CCI was determined to correct for the severity of comorbidities. Charlson et al., [43] defined numerous clinical conditions through reviewing hospital charts and assessed their relevance in the prediction of 1-year mortality. Based on patient medical history prior to the time of diagnosis, weights were assigned to 17 comorbidities and then summed. We used an algorithm converted to ICD-10 [44]. The CCI score was calculated based on the observation period of 1 year before the first health examination date.

### 4.3. Assessment of First-Step Analgesic Use

This study explored the inhibitory effect of cancer occurrence through medication compliance (regularity) based on WHO 3-step ladder analgesics, which are indicated for pain of various causes, such as occupational stress, anxiety/depression (mood disorder), diabetes, hypertension, and obesity, etc. [45,46]. WHO first-step analgesics are non-opioid drugs prescribed to control pain. Non-opioid analgesics are effective for inflammatory conditions of somatic pain and acute pain, and include aspirin (acetyl salicylic acid), acetaminophen, and nonsteroidal anti-inflammatory drugs (NSAIDs) [47].

We approached this study from the perspective of medication compliance (high compliance with pain control; regular intake/low compliance with pain control; irregular intake) for the purpose of controlling for pain caused by various causes (before cancer occurrence).

In this respect, non-opioid analgesics were evaluated comprehensively without classification according to the expression of cyclooxygenase (COX). The first-step analgesics considered included acetaminophen, aspirin, piroxicam, diclofenac, celecoxib, ibuprofen, naproxen, mefenamic acid, ketoprofen, dexibuprofen, and others.

Analgesic use was determined by extracting the prescription history corresponding to first-step analgesics for 2 years from the baseline. Regular use was defined as prescription for >15 days per month for >6 months [48]. No use (never) was defined as no prescription history.

### 4.4. Statistical Analysis

The differences in economic activity, health behavior, CCI, and first-step analgesic use in each working-age group were examined using the chi-square test. We calculated HRs with 95% CIs for patient risk of developing cancer based on economic activity (NPEA or PEA) and analgesic use (Model 1). Additionally, we determined the risk of cancer by specific age group (Model 2) using a proportional hazards regression model. All statistical analyses were performed using the statistical package R version 4.2 (R Core Team, Vienna, Austria).

## 5. Conclusions

The results of this study showed that the risk of breast cancer was high among those who engaged in economic activity, had a high income, and no history of exercise in all working-age groups. Notably, participants in the Middle-aged group (40~49 years) who engaged in economic activity had the highest HR of breast cancer development. Therefore, since women aged 40~49 years account for a major portion of the economic population, policies are required to reduce their burden of childcare and unstable employment, and ameliorate their health behavior. Particularly, attention should be paid to a lack of exercise among these women. Furthermore, regular analgesic use may be beneficial for inhibiting cancer development in the Young-aged group (24~39 years). Our results suggest that the group at high-risk of comorbidity may benefit from the regular use of analgesic, which may prove to be a useful strategy for breast cancer prevention in the Young-aged group.

## Figures and Tables

**Table 1:** Baseline characteristics of the study population.

Variables	Descriptions	Young-Aged (25~39)		Middle-Aged (40~49)		Senior-Aged (50~64)		*x* ^*2*^	*p*
		*N*	%	*N*	%	*N*	%		
Insurance type									
	Members ^(a)^	950	39.7	1125	43.9	500	28.0	475.50	0.000
	Dependents ^(b)^	815	34.1	825	32.2	940	52.7		
	Self-employed	220	9.2	400	15.6	295	16.5		
	Employee	405	16.9	210	8.2	50	2.8		
Income									
	Low (1~3)	695	29.1	817	31.9	611	34.2	49.85	0.000
	Middle (4~6)	875	36.6	743	29.0	495	27.7		
	High (7~10)	820	34.3	1000	39.1	679	38.0		
Body mass index (BMI)									
	<25	1820	76.2	1756	68.6	1026	57.5	164.75	0.000
	≥25	570	23.8	804	31.4	759	42.5		
Total cholesterol									
	<240	2225	93.1	2220	86.7	1409	78.9	178.49	0.000
	≥240	165	6.9	360	14.1	376	21.1		
Alcohol intake									
	Never	1531	64.1	1855	72.5	1547	86.7	267.80	0.000
	Ever	859	35.9	705	27.5	238	13.3		
Smoking									
Never		2161	90.4	2347	91.7	1686	94.5	22.99	0.000
Ever		229	9.6	213	8.3	99	5.5		
Exercise									
	Never	1011	42.3	1242	48.5	945	52.9	48.15	0.000
	Ever	1379	57.7	1318	51.5	840	47.1		
Charlson Comorbidity Index (CCI)									
	0	1515	63.4	1392	54.4	639	35.8	517.46	0.000
	1	573	24.0	641	25.0	468	26.2		
	2	208	8.7	301	11.8	293	16.4		
	≥3	94	3.9	226	8.8	385	21.6		
Analgesics use									
	Never	536	22.4	600	23.4	299	16.8	201.95	0.000
	Non-regular	1572	65.8	1630	63.7	1013	56.8		
	Regular	282	11.8	330	12.9	473	26.5		

^(a)^ Members: family members of self-employed ^(b)^ Dependents: dependents of insured employees.

**Table 2:** Hazard ratios (HRs) associated with cancer development in a cohort of 6735 patients.

	Variables	HR ^(a)^	95% CI ^(b)^	*p*
Economic activity				
	Not participating	1.000		
	Participating	1.542	1.345–1.768	0.000 ***
Income				
	Low (1~3)	1.000		
	Middle (4~6)	1.673	1.419–1.973	0.000 ***
	High (7~10)	3.089	2.647–3.605	0.000 ***
Body mass index (BMI)		0.990	0.975–1.006	0.478
Total cholesterol		0.998	0.997–0.999	0.007 **
Alcohol intake				
	Never	1.000		
	Ever	0.967	0.853–1.096	0.600 **
Smoking				
	Never	1.000		
	Ever	0.638	0.497–0.819	0.000 ***
Exercise				
	Never	1.000		
	Ever	0.836	0.751–0.931	0.001 **
Charlson Comorbidity Index (CCI)				
	0	1.000		
	1	0.993	0.870–1.134	0.919
	2	1.123	0.946–1.343	0.186
	≥3	1.113	0.921–1.345	0.268
First-step analgesics use				
	Never	1.000		
	Non-regular	0.905	0.785–1.043	0.168
	Regular	0.748	0.614–0.912	0.004 **

^(a)^ HR: hazard ratio, ^(b)^ CI: confidence interval, ** *p* > 0.05, *** *p* > 0.001.

**Table 3:** Risk of breast cancer by specific working-age group.

Model 1 ^(a)^		Young-Aged (25~39)			Middle-Aged (40~49)			Senior-Aged (50~64)		
		HR ^(b)^	95% CI ^(c)^	*p*	HR	95% CI	*p*	HR	95% CI	*p*
Economic activity										
	Not-participating	1.000			1.000			1.000		
	Participating	1.482	1.185–1.854	0.001 **	1.700	1.361–2.124	0.000 ***	1.386	1.049–1.833	0.022 **
Income										
	Low (1~3)	1.000			1.000			1.000		
	Middle (4~6)	1.731	1.314–2.281	0.000 ***	1.763	1.334–2.330	0.000 ***	1.556	1.142–2.120	0.005 **
	High (7~10)	3.161	2.415–4.138	0.000 ***	3.504	2.712–4.529	0.000 ***	2.503	1.895–3.306	0.000 ***
Charlson Comorbidity Index (CCI)										
	0	1.000			1.000			1.000		
	1	1.234	0.994–1.534	0.057	0.969	0.785–1.196	0.767	0.730	0.551–0.968	0.029 **
	2	1.555	1.149–2.105	0.005 **	0.815	0.603–1.100	0.181	1.000	0.739–1.354	0.999
	≥3	2.095	1.422–3.086	0.000 ***	0.976	0.703–1.356	0.886	0.823	0.606–1.118	0.213
First-step analgesics use										
	Never	1.000			1.000			1.000		
	Non-regular	0.915	0.723–1.157	0.458	0.912	0.728–1.142	0.423	0.912	0.678–1.227	0.542
	Regular	0.611	0.427–0.875	0.007 **	0.929	0.670–1.288	0.659	0.741	0.517–1.061	0.100 *

^(a)^ Adjusted for income (low, middle, high), BMI, total cholesterol, alcohol intake, smoking and exercise (never, ever). ^(b)^ HR: hazard ratio, ^(c)^ CI: confidence interval. * *p* > 0.1, ** *p* > 0.05, *** *p* > 0.001, Model 1: Analysis results of the inhibitory effect of first-step analgesics on cancer occurrence.

## Data Availability

Data is contained within the article and Supplementary Materials.

## Supplementary Materials

The following supporting information can be downloaded at: https://www.mdpi.com/article/10.3390/ph16020323/s1, Table S1. HRs associated with cancer development in a cohort of 6735 patients for acetaminophen and NSAIDs. Table S2. Risk of breast cancer by specific working-age group for acetaminophen and NSAIDs. It was classified into acetaminophen, which has no anti-inflammatory effect (non-NSAIDs), but which has analgesic and antipyretic effects, and NSAIDs, which have anti-inflammatory effects as indications. NSAIDs differ in COX 1 and 2 inhibitory effects depending on their mechanism of action and dose, but they are first-step analgesics, excluding acetaminophen, such as aspirin, ibuprofen, dexibuprofen, and celecoxib, etc.
