# Supplementary material for: Association between WHO First-Step Analgesic Use and Risk of Breast Cancer in Women of Working Age
Source: Pharmaceuticals (Basel). 2023 Feb 20;16(2):323. doi: 10.3390/ph16020323 (PMC9961524; doi:10.3390/ph16020323)
Supplement: Supplementary file 1 [file pharmaceuticals-16-00323-s001.zip › pharmaceuticals-2127355-supplementary.pdf]

Supplementary Table S1. HRs associated with cancer development in a cohort of 6735 patients for acetaminophen and NSAIDs.

Variables	HR[(a)]	95% CI[(b)]	*p*
Economic activity			
Not participating	1.000		
Participating	1.539	1.343–1.764	0.000 ***
Income			
Low (1~3)	1.000		
Middle (4~6)	1.673	1.419–1.973	0.000 ***
High (7~10)	3.083	2.642–3.598	0.000 ***
Body mass index (BMI)	0.995	0.979–1.011	0.515
Total cholesterol	0.998	0.997–1.000	0.008 **
Alcohol intake			
Never	1.000		
Ever	0.966	0.852–1.094	0.585
Smoking			
Never	1.000		
Ever	0.642	0.500–0.824	0.000 ***
Exercise			
Never	1.000		
Ever	0.835	0.750–0.930	0.001 **
Charlson Comorbidity Index (CCI)			
0	1.000		
1	0.988	0.865–1.129	0.862
2	1.115	0.939–1.324	0.215
≥3	1.119	0.925–1.354	0.246
Acetaminophen			
Never	1.000		
Non-regular	0.960	0.851–1.083	0.507
Regular	0.813	0.651–1.015	0.067 *
Non-steroidal anti-inflammatory drugs (NSAIDs)			
Never	1.000		
Non-regular	0.905	0.861–1.093	0.621
Regular	0.748	0.570–1.010	0.058 *

[(a)]HR: hazard ratio, [(b)]CI: confidence interval. * $p > 0.1$, ** $p > 0.05$, *** $p > 0.001$.

Supplementary Table S2. Risk of breast cancer by specific working-age group for acetaminophen and NSAIDs.

Model 2[(a)]	Young-aged (25~39)			Middle-aged (40~49)			Senior-aged (50~64)		
	HR[(b)]	95% CI[(c)]	*p*	HR	95% CI	*p*	HR	95% CI	*p*
**Acetaminophen**									
Never	1.000			1.000			1.000		
Non-regular	1.007	0.824–1.232	0.943	0.953	0.785–1.157	0.626	0.913	0.717–1.163	0.461
Regular	0.687	1.392–3.018	0.063 *	0.915	0.630–1.330	0.643	0.809	0.546–1.199	0.291
Non-steroidal anti-inflammatory drugs (NSAIDs)									
Never	1.000			1.000			1.000		
Non-regular	0.945	0.777–1.151	0.576	1.075	0.888–1.301	0.457	0.900	0.704–1.149	0.397
Regular	0.732	0.338–1.586	0.430	0.707	0.388–1.288	0.257	0.797	0.539–1.180	0.257

[(a)]adjusted for income (low, middle, high), BMI, total cholesterol, alcohol intake, smoking and exercise (never, ever). [(b)]HR: hazard ratio, [(c)]CI: confidence interval. * $p > 0.1$, Model 2: Analysis results of the inhibitory effect of acetaminophen and NSAIDs on cancer occurrence.
